# Risk factors and mortality rates for children co-infected with HIV and TB in Ethiopia: a systematic review and meta-analysis

**DOI:** 10.1093/inthealth/ihaf085

**Published:** 2025-08-21

**Authors:** Fassikaw kebede Bizuneh, Tsehay Kebede Bizuneh, Biruk Beletew Abate, Atitegeb Abera Kidie, Gataye Tizazu Biwota, Tilahun Gizaw Ayenew

**Affiliations:** College of Medicine and Health Science, Debre Markos University, P.O. Box 269, Ethiopia; Department of Geography, Colleges of Social Science, Bahir Dare University, P.O. Box 72, Bahir Dare, Ethiopia; Department of Nursing, College of Health science, Woldia University, P.O. Box 400, Woldia, Ethiopia; Department of Nursing, College of Health science, Woldia University, P.O. Box 400, Woldia, Ethiopia; College of Medicine and Health Science, Debre Markos University, P.O. Box 269, Ethiopia; Department of Psychiatry, College of medicine and Health science, Jigjiga University, P.O. Box 1020, Jigjiga, Ethiopia

**Keywords:** children, Ethiopia, HIV, mortality, pediatrics, predictors, TB

## Abstract

This systematic review and meta-analysis aimed to identify risk factors and mortality rates in HIV and TB co-infected children in Ethiopia. An electronic literature search was conducted using multiple databases, including PubMed, Medline, Web of Science, African Journal Online, Google Scholar and university research repositories for gray literature. Weighted inverse variance random-effects meta-regression was employed to calculate pooled mortality rates, utilizing Stata/SE-17 for analysis. The meta-analysis included six eligible studies, encompassing a total of 2025 co-infected children. Among these, 238 deaths were reported over 1670.6 person-years. This made the crude mortality rate 11.74% (95% CI 11.49 to 16.12%) with an incidence of 1.5 deaths (95% CI 1.17 to 1.89) per 100 person-years. Factors including WHO stages III and IV (4.34, 95% CI 2.25 to 8.36), poor antiretroviral therapy (ART) adherence (3.11, 95% CI 2.04 to 4.15), missed isoniazid preventive therapy (IPT) (3.07, 95% CI 1.52 to 6.23) and low hemoglobin levels of ≤10 mg/dl (2.84, 95% CI 2.02 to 3.99) were predictors compared with their counterparts.This review reveals an unacceptably high pooled incidence of mortality among HIV and TB co-infected children in Ethiopia. Therefore, implementing systematic screenings for IPT, enhancing ART adherence counseling and addressing anemia through early treatment are critical for preventing premature deaths.

Protocol registration in Prospero = CRD42024502038

## Introduction

HIV and TB are the two main deadly pandemics from infectious diseases.^1,2^ There is a strong synergy between HIV infection and TB. People living with HIV (PLHIV) are at a high risk of morbidity and mortality from TB, as HIV infection is the primary risk factor for the incidence of active TB.^3^ Both diseases interact in a complex and synergistic manner. HIV infection weakens the immune system and increases susceptibility to active TB incidence in the lung.^4^ TB infection also accelerates the progression of advanced HIV infection by initiating an immune response that can boost HIV replication and viral load.^5,6^ The immune activation caused by TB leads to inflammation and immune system dysfunction, which can affect the efficacy of antiretroviral drugs.^7^ According to the 2023 Global TB Report, about 1.3 million (12% of 10.6 million) new TB cases were children aged 0–14 y.^2^ Of those, 14.3% were co-infected with HIV and TB.^2^ About 214 000 patients in 2020, and 187 000 in 2021, died during TB and HIV co-infections.^2,8^ Despite advances in diagnostics and the widespread availability of antibiotics, TB infection remains responsible for 40% of deaths, 18% of hospitalizations and 25% of case fatalities for PLHIV.^9^

Previous study findings suggested that TB and HIV co-infection mortality varied across developed and developing countries.^10,11^ One systematic review and meta-analysis (SRM) reported that the mortality rate attributable to TB was as high as 24.9% in adults and 30.1% in children.^12^ A cluster randomized trial study in South Africa indicated that the mortality rate among patients co-infected with TB and HIV was 10.1 per 100 person-years.^13^ A study in Malaysia reported that the mortality rate for TB patients co-infected with HIV was 23.3%.^14^ In sub-Saharan Africa (SSA), the mortality rate for dual TB and HIV co-infection ranges from 10% to 29.8% and varies within each country.^9^ Evidence from Tanzania suggested that the incidence of mortality among patients with TB and HIV was 57.8 per 1000 person-years.^15^ By contrast, the reported death rate during TB and HIV co-infection in Uganda was lower (6.38%) after antiretroviral therapy (ART) was initiated.^16^ In Ethiopia, the true burden of childhood TB is often underestimated.^7,17^ The regional healthcare disparities and sociodemographic differences among caregivers increase the number of premature deaths in children during co-infections.^6^

Several predicting factors^3,17–21^ have been identified as contributors to mortality during co-infection for children living with HIV/AIDS: missed isoniazid preventive therapy (IPT),^22,23^ poor ART adherence^11,14^ and immune reconstitution inflammatory syndrome^9,24^ were predictors for premature death. Recent findings suggest that a CD4 count of ≤200 cells/mL and a viral load of ≥400 copies/mL during TB co-infection in children with HIV are proxy indicators for premature death.^14,25,26^ In Ethiopia, efforts have been made to reduce morbidity and mortality during co-infections through community-based case training, provision of free ART and anti-TB drugs and free provision of IPT, resulting in significant progress being recorded.^17,27^ However, in Ethiopia, there is a lack of aggregated mortality data of co-infected children. Therefore, an SRM was conducted for estimating the incidence rate and predictors of mortality among ART-taking TB and HIV co-infected children in Ethiopia.

## Methods

### Reporting and protocol registration

This SRM was conducted following Preferred Reporting Items for Systematic Reviews and Meta-Analyses (PRISMA) guidelines (Supplementary Data 1)^28^ and was prospectively registered on Prospero with the registration number CRD42024502038.

### Data-searching strategy

Primary studies in English and other languages were retrieved from internationally reputable electronic databases in Ethiopia. PubMed, Medline, Web of Science, African Journal Online, Google Scholar and university research repositories were searched to find pertinent articles from 16 October to 21 November 2024. We employed free text, keywords and Medical Subject Headings (MeSH) for searching eligible studies from electronic databases. The search terms included keywords, free texts and MeSH terms used to access the eligible studies as follows: ‘Incidence’, ‘Incidence’ [Mesh], ‘incidence rate’, ‘incidence density’, magnitude, ‘Survival’ [Mesh], ‘Mortality’ [Mesh], predictors, ‘TB-HIV co-infection’, ‘Child’ [Mesh], ‘pediatrics’ [Mesh], ‘children’ [Mesh], ‘Adolescent’ [Mesh] and Ethiopia.

The search terms and keywords were combined using ‘AND’ and ‘OR’ Boolean operators to yield sufficient and appropriate search results (Supplementary Data 2). This SMR followed the PICO—Population, Intervention, Comparison and Outcomes—framework to evaluate the eligibility of published articles for developing evidence-based clinical research. This is outlined as Population (P): children co-infected with TB and HIV in Ethiopia; Intervention (I): HIV-infected children who had undergone ART; Comparison (C): children co-infected with TB and HIV; and Outcome (O): the primary outcome of interest was TB-associated death while under care.

### Eligibility: criteria of articles

#### Inclusion and exclusion criteria

This review encompasses all published observational studies, regardless of language and publication date, conducted in Ethiopia and focusing on co-infected children aged ≤15 y who were considered eligible. However, papers that did not report the outcomes of interest, as well as those lacking the full text, were excluded from the analysis.

#### Outcome ascertainments

The dependent variable is death during TB and HIV co-infected children. This was calculated by extracting mortality proportions from each eligible study and dividing by the person per months of risk observation using Pro-Command in STATA version 17 (StataCorp LLC, Texas, USA).

#### Article quality assessment and appraisal

The methodological quality of the retained publications was assessed by two authors (FKB and GTB) using the Joanna Briggs Institute (JBI) checklist for cohort studies (Supplementary Data 3).^29^ The tool has the following criteria for each study: appropriate statistical analysis, strategies to address incomplete follow-up, sufficient follow-up time, valid and reliable measurement of outcomes, participants free of the outcome at the beginning of the study, identification of confounding factors and strategies to reduce missing dates. Questions that meet these criteria are labeled as ‘1’, while those that do not meet the criteria are labeled as ‘0’. During labeling of the eligible studies, scoring ≥50% on the quality assessment tools was considered to be a low risk/high quality score. Studies scoring <49% on the quality assessment checklist were categorized as high risk/low quality based on the average positive score of the JBI checklist.

#### Data extraction and screening process

Two authors (FKB and GTB) screened the article titles and abstracts against the inclusion and exclusion criteria. Four reviewers (FKB, AAK, BBA and TGA), according to predetermined exclusion and inclusion criteria from 16 October to 21 November 2024, then extracted relevant data. Any disagreements were resolved through discussion through involvement of a third author (TKB) to address uncertainties. Two authors (FKB and GTB) were assigned to remove unrelated studies based on their titles and abstracts. Discrepancies between the authors were settled by discussion. Author group, publication year, study region, study design, study setting, sample size, outcome, response rate, incidence rate of mortality and predictors' effect size were extracted to a Microsoft Excel spreadsheet before analysis was started.

#### Handling of missing dates

During meta-analysis reports, missing data were addressed using the complete case analysis method. Given that the missing values were <5%, there was no indication to carry out single or multiple imputations.^30^

#### Software and statistical analysis

Using EndNote version 8.1, all potentially suitable published article citations were exported and duplications were removed during the screening process. Microsoft Excel (Meta-XL) version 5.3 was employed to extract relevant data from included studies before they were exported to STATA version 17 statistical software for further analysis.^31^ The pooled mortality rate among children co-infected with TB and HIV and its predictors was estimated using weighted inverse variance random-effects meta-regression combined with DerSimonian–Laird model weight.^32,33^ Descriptive statistical results are presented in tables and funnel plots.

#### Publication of bias and sensitivity analysis

The presence of heterogeneity between included studies was evaluated using the Cochrane Q-test and I^2^ statistics. Publication bias was checked through graphical inspection (funnel plot) and quantitatively using Egger's weighted regression test.^32,33^ In addition, we performed a leave-one-out sensitivity analysis to confirm a study with a biased direction of pooled estimates of Beggs and Eggers tests.^30^ Subgroup analysis was performed to adjust random variation in the presence of significant heterogeneity between primary studies.

## Results

### Screening of included studies

**Figure 1.**
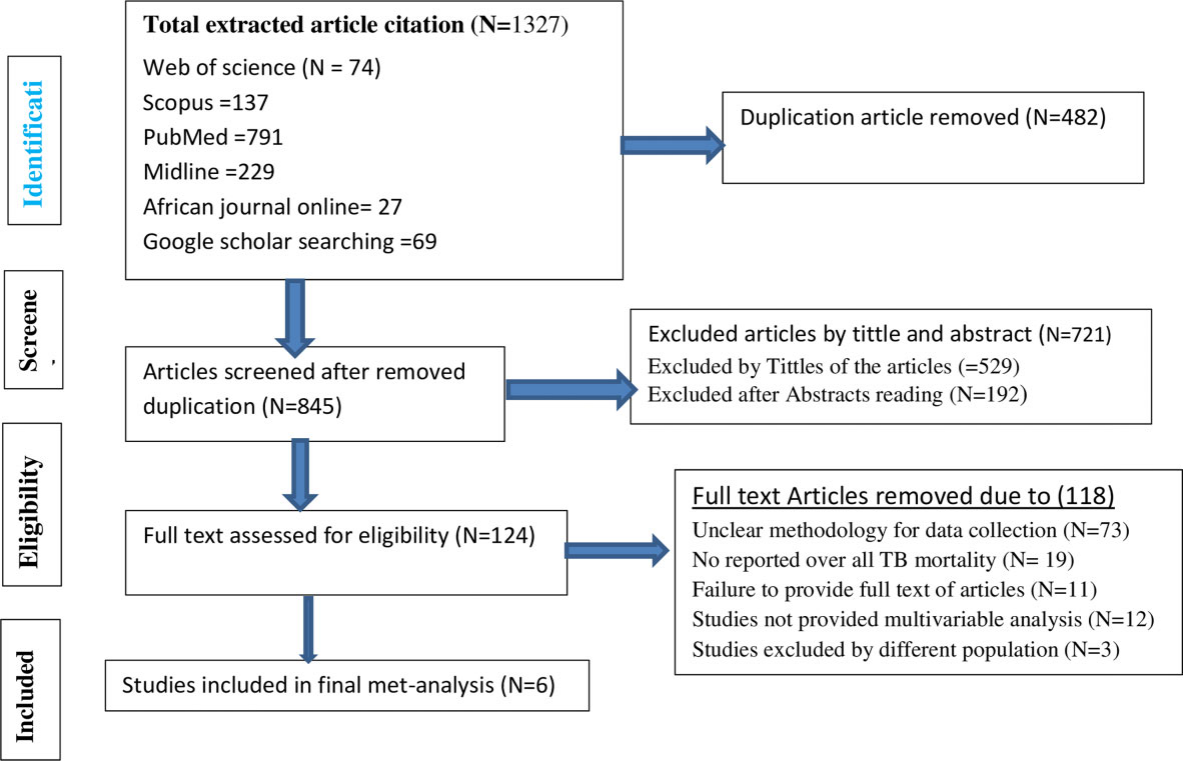
Article selection flowchart by PRISMA for children co-infected with TB and HIV in Ethiopia.

Our search using the prespecified strategies resulted in 1327 published articles. During the initial article screening process, 482 duplicates were removed. After thoroughly reviewing the titles and abstracts of 845 articles, we excluded 721 articles that did not meet the criteria. During the full-text review, we evaluated 124 publications, of which 118 articles were excluded for various reasons, including unclear methodology (N=73), insufficient data collection and reporting format (N=19), inaccessibility of full texts (N=11), absence of multivariable predictors (N=12) and differences in the population (N=3) (Figure 1).

### Descriptive reports of included studies

In this SRM, six primary studies^34–39^ were included to estimate the pooled incidence of mortality rate among children co-infected with TB and HIV. The included articles were from three regions: there were three from Amhara,^34,35,39^ two from the Southern Nation Nationalities Region (SNNR)^36,37^ and one from Tigray.^38^ The study-specific sample sizes ranged from 227 in Debre Tabor^35^ to 498 in Woldia^39^ hospitals, both located in the Amhara region. On the other hand, the estimated effect sizes of mortality from the individual studies ranged from 47 (17.2%)^36^ to 35 (12.3%)^37^ multi-sites of the SNNR. All of the studies employed a retrospective cohort and were conducted in a hospital set-up. The follow-up period for the included studies ranged from 30 June 2021 to 30 September 2023.^34–39^ Regarding article quality, all the studies were of high quality, scoring ≥80%.^34–39^ The mean age of participants ranged from 6.6 (±3.5)^34^ to 9.14 (±3.7) y^39^ (Table 1).

**Table 1.** Characteristics of included articles for TB-associated death for PLHIV in Ethiopia

Authors	Year	Region	Design	Population	Setting	Mean age, y	Sample size	Number of deaths	Study period	PPM PPY	Death proportion	Quality
Jifare Gemechu et al.^31^	2022	SNNR	RC	Children	HT	7.1 (±3.7)	284	35	1 January 2009 to 30 December 2019	4016.1	0.123 239 437	Low risk
										334.6		
Zinabu Dawit et al.^14^	2021	SNNR	RC	Children	HT	8.9 (±3.5)	274	47	1 January 2009 to 31 December 2018	3331	0.171 532 847	Low risk
										277.6		
Ermias Sisay et al.^19^	2021	Amhara	RC	Children	HT	6.70	227	39	1 March 2014 to 12 January 2021	2388	0.171 806 167	Low risk
										199		
Jemberu Nigussie et al.^47^	2021	Tigray	RC	Children	HT	8 (SD 4–13)	253	38	1 January 2008 to 30 December 2018	3682	0.150 197 628	Low risk
										306.8		
Kindalem Attale et al.^30^	2018	Amhara	RC	Children	HT	6.6 (±3.5)	271	39	1 February 2005 to 1 March 2017	4412	0.143 911 439	Low risk
										367.6		
Dejen Tsegaye et al.^41^	2023	Amhara	RC	Children	HT	9.14 (±3.7)	498	40	1 November 2015 to 30 December 2021	2222	0.080 321 285	Low risk
										185.2		

PLHIV: people living with HIV; PPM: person per month risk observation; PPY: persons per year; RC: retrospective cohort; SNNR: South Nation Nationalities Region.

### Pooled incidence density rate of TB and HIV co-infected mortality

This study comprised 2025 TB and HIV co-infected children aged ≤15 y. During mortality screening, 238 deaths were reported within 1670.6 persons per year of risk observation.

**Figure 2.**
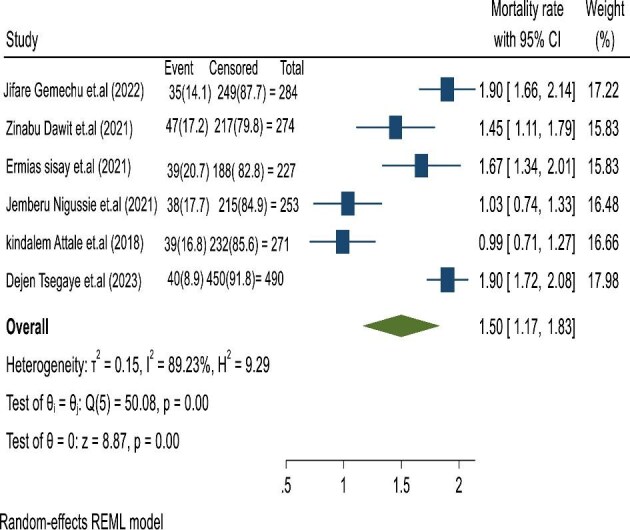
Pooled incidence of mortality rate for children co-infected with TB and HIV in Ethiopia.

We found a crude mortality rate of 11.74% (95% CI 11.49 to 16.12%) and an incidence of 1.5 (95% CI 1.17 to 1.89) cases per 100 person-years (Figure 2).

### Handling heterogeneity and subgroup analysis

To address the observed heterogeneity among studies, subgroup and sensitivity analyses were conducted based on follow-up time, study region, sample size and study setting. The mortality rate for children during co-infection was higher in the SNNR (1.69%) compared with the Tigray region (1.03%). Subgroup analysis concerning sample size and mortality during co-infection was elevated for pooled studies with sample sizes of ≥260 participants (1.57%) compared with sample sizes of <260 (1.35%). Interestingly, consistent mortality rates were found across different follow-up times in pooled studies with rates of 1.45% for ≤120 mo and 1.52% for >120 mo (Table 2).

**Figure 3.**
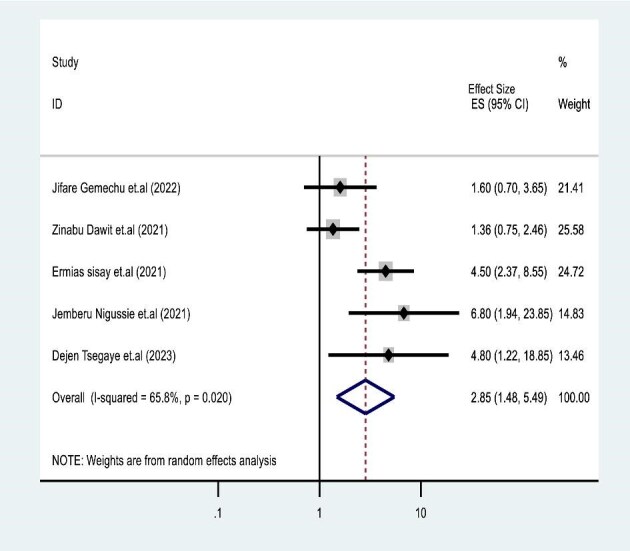
Forest plots for association with WHO clinical stages III and IV and mortality in co-infected children.

**Table 2.** Subgroup analysis of mortality in children co-infected with TB and HIV after ART in Ethiopia

	Category		Included studies	IR per 100 PPMO (95% CI)	Heterogeneity (I^2^, p value)
1	Region	Amhara	3	1.53	92.32, 0.001
		SNNR	2	1.69	77.73, 0.03
		Tigray	1	1.03	89.3, 0.001
2	Follow-up time (y)	≥10	2	1.45	95.23, 0.001
		<10	4	1.52	86.2, 0.001
3	Sample size	≤260	2	1.35	87.2, 0.01
		>260	4	1.57	91.4, 0.001

ART: antiretroviral therapy; IR: incidence rate; PPMO: person per month observation; SNNR: South Nation Nationalities Region.

### Predictors for mortality during TB and HIV co-infected mortality

To identify risk factors for mortality during TB co-infection in HIV-infected children, significant categorical risk factors were pooled from each study. After employing weighted inverse variance random-effects meta-regression, four statistically significant variables were identified, consisting of advanced WHO clinical stages III and IV, poor/fair ART adherence, missed IPT and low levels of hemoglobin (Hgb), as presented in Table 3.

**Table 3.** Factors associated with TB and HIV co-infection mortality in children with HIV

Categorical variable	Included studies	HR	95% CI		Pooled HR	I^2^	p
WHO	Jifare Gemechu et al.^31^	1.6	0.71	3.7	2.85 (1.48 to 5.49)	65.4%	0.02
	Zinabu Dawit et al.^14^	1.36	0.74	2.43			
	Ermias Sisay et al.^19^	4.5	2.39	8.63			
	Jemberu Nigussie et al.^47^	6.8	2.01	24.6			
	Dejen Tsegaye et al.^41^	4.8	1.2	18.56			
ART adherence	Jifare Gemechu et al.^31^	1.25	1.25	7.46	3.11 (2.04 to 4.11)	20.2%	0.20
	Zinabu Dawit et al.^14^	2.19	2.19	12.7			
	Ermias Sisay et al.^19^	0.799	0.799	3.5			
	Jemberu Nigussie et al.^47^	1.39	1.39	10.79			
	Kindalem Attale et al.^30^	1.7	1.7	7.8			
CPT	Zinabu Dawit et al.^14^	1.5	1.25	7.46	2.39 (0.84 to7.2)	0.01	0.09
	Ermias Sisay et al.^19^	3.8	2.19	12.7			
	Jemberu Nigussie et al.^47^	1.79	0.799	3.5			
	Dejen Tsegaye et al.^41^	4.1	1.39	10.79			
IPT	Jifare Gemechu et al.^31^	0.55	0.29	2.6	3.07 (1.52 to 6.23)	76.1	0.01
	Zinabu Dawit et al.^14^	7.27	5.5	13.89			
	Ermias Sisay et al.^19^	2	0.78	5.6			
	Jemberu Nigussie et al.^47^	3.7	1.5	10.5			
	Kindalem Attale et al.^30^	3.87	1.6	9.2			
	Dejen Tsegaye et al.^41^	4.8	1.6	10.3			
Level of hemoglobin	Jifare Gemechu et al.^31^	3.6	1.39	9.56	2.84 (2.02 to 3.99)	0.1	0.7
	Zinabu Dawit et al.^14^	2.69	1.52	5.64			
	Ermias Sisay et al.^19^	7.6	0.53	13.73			
	Jemberu Nigussie et al.^47^	3.75	1.5	13.6			
	Kindalem Attale et al.^30^	2.6	1.24	5.34			
	Dejen Tsegaye et al.^41^	2.2	1.2	4.6			

ART: antiretroviral therapy; CPT: cotrimoxazole preventive therapy; IPT: isoniazid preventive therapy.

### Effects of WHO stages III and IV on TB and HIV co-infected mortality

Five studies^35–39^ were used to test the association of advanced WHO stages III and IV with TB co-infection mortality of HIV-infected 1527 participant children. In the final review of this SRM, TB co-infection and advanced WHO stages III and IV were associated with a fourfold increased mortality risk (pooled HR 4.34; 95% CI 2.25 to 8.36) with significant heterogeneity (I²=95.50%, p=0.001) (Figure 3).

### Effect of poor ART adherence on TB and HIV co-infected mortality

**Figure 4.**
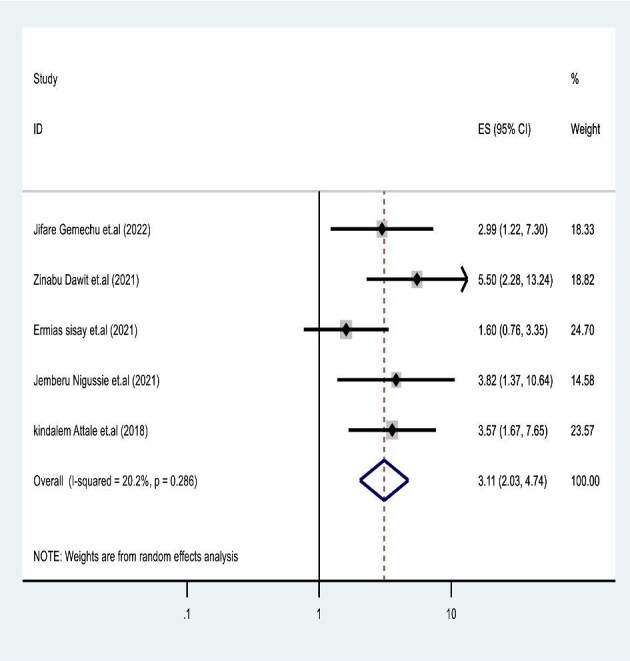
Forest plot showing poor ART adherence and mortality incidence in children co-infected with TB and HIV. ART: antiretroviral therapy.

A total of five individual studies^34–38^ were used to assess the association between poor ART adherence and mortality in 1487 children co-infected with TB and HIV. The report indicated a strong correlation of premature deaths in children with poor or fair ART adherence during co-infection. The final random-effects model analysis revealed that children with poor and fair ART adherence had an HR of 3.11 (95% CI 2.04 to 4.15) for mortality compared with those with good adherence (Figure 4).

### Effects of missed IPT on TB and HIV co-infected mortality

Six studies^34–39^ were used to investigate the association between missed IPT and deaths due to TB incidence following ART initiation. All the included studies reported a significant risk of TB-associated death and missed IPT after ART initiation among children. Random-effects model analysis indicated that patients co-infected with TB and HIV who had missed IPT experienced a threefold hazard of death (pooled HR=3.07; 95% CI 1.52 to 6.23) with significant heterogeneity (I^2^=96.6%).

### Effects of low Hgb level on TB and HIV co-infected mortality

Low Hgb levels and TB-associated mortality among HIV-infected children were assessed using a pooled risk ratio from six studies.^34–39^ The final SRM regression finding reported that children with Hgb≤10 mg/dl had a threefold higher risk of death during TB co-infection compared with Hgb>10 mg/dl (pooled HR=2.84; 95% CI 2.02 to 3.99).

### Impacts of missed cotrimoxazole preventive therapy on TB and HIV co-infected mortality

The association between missed cotrimoxazole preventive therapy (CPT) and TB and HIV co-infected mortality was examined using 1229 participants from five studies.^34–36,38,39^ However, the final meta-regression report indicated that missed CPT had no significant correlation with TB and HIV co-infected mortality of children.

### Small study effects and publication bias assessment

We tested for publication bias using graphical and statistical tests. In graphical assessments, asymmetric distributions of articles were observed in the funnel plot upon visual inspection. In addition, we performed quantitative Egger regression to investigate small study effects: the statistical test revealed that there was no significant publication bias, with an intercept of slope at 1.081 and p=0.074 with a bias co-efficient of -5.073 at p=0.214.

## Sensitivity analysis

We also conducted a leave-one-out sensitivity analysis to investigate the influence of individual studies, and the results indicated that no single study significantly influenced the overall estimation rate (incidence density rate=1.41%).

## Discussion

The rate was found to be 1.5 (95% CI 1.17 to 1.89) cases per 100 person-years among TB and HIV co-infected children in Ethiopia. This description is higher than those previously reported in Congo^40^ and China.^11^ Possible reasons for this could be disparities in healthcare services, as well as differences in socioeconomic status, study populations and methodologies.^41^ However, the finding was lower than those reported by a systematic review conducted in low- and middle-income countries (10.8%^12^) and in Ethiopia (3.54%^17^). These discrepancies may stem from sociodemographic factors, differing study periods and cultural disparities. Another factor could be substandard healthcare and TB treatment guidelines during co-infection treatment. Conversely, the estimated mortality rate was consistent with those reported in Finland^42^ and in SSA.^10^ This may be linked to the positive effects of early initiation of ART in Ethiopia and some SSA countries (e.g. Kenya, Uganda and Tanzania) for children aged ≤15 y resulting in better health outcomes.^21^

The subgroup analysis by region revealed that the highest mortality rate was observed in studies from the SNNR (1.69%) compared with those from Amhara (1.53%) and Tigray (1.03%). This variance may be due to the inclusion of a limited number of studies for rate estimation and differences in sample sizes. On the other hand, fragmentation of the SNNR into three subregions during the COVID-19 pandemic led to reduced healthcare provision in each area, affecting the quality of health services for PLHIV.^43^

Unlike previous study findings^17,21^ in Ethiopia, TB-associated mortality rates for HIV co-infected children remained consistent, irrespective of follow-up duration (≤10 y: 1.45%; >10 y: 1.52%). This might be due to the rapid disease progression, co-infection complications and inconsistent healthcare access across each region.^17,18^ It might also be directly linked to a prolonged conflict spanning nearly 4 y in Ethiopia between the Tigray, Afar and Amhara regions that has led to 94% of medical equipment being stolen and destruction in each region, with 92% of HIV care services being severely disrupted, including care provision for children.^25^ The disruption to healthcare services has impacted care for PLHIV and increased co-infection mortality in each region.

The findings of this review indicate that children co-infected with TB and HIV in advanced WHO clinical stages III and IV experienced fourfold hazards of death compared with other groups. This is consistent with previous findings in Wolaita Sodo,^44^ Gondar,^17^ Tanzania,^45,46^ South Africa^9^ and SSA,^10^ as well as a global systemic review.^12^ As HIV progresses to later clinical stages, patients become more vulnerable to deadly opportunistic infections such as TB due to compromised immunity, leading to reduced survival rates in co-infected individuals.^4^ On the other hand, evidence suggests that in children with HIV at advanced clinical stages (III and IV), there is a sharp decline in cellular immunity and rapid viral replication, indirectly indicating a significant decrease in CD4 counts below the threshold, which serves as a proxy indicator for premature death during co-infection.^4,7^

Consistent with previous SMR findings^4,6,47^ and results from Kazakhstan,^23^ co-infected children who missed IPT experienced a significant risk of mortality. In this review, TB and HIV co-infected children who missed IPT had a threefold increased risk of death compared with those who received IPT. A primary study^41^ and a meta-analysis^47^ in Ethiopia reported that IPT can reduce the risk of latent TB reactivation and prevent 90% of TB-associated deaths when administered concurrently with ART. However, drug complexity, side effects and the forgetfulness of caregivers reduced the completion rates of IPT and increased susceptibility to TB infection among children.

The hazard of death for children with TB co-infected with HIV with Hgb≤10 g/dl were increased threefold compared with those groups with Hgb>0 mg/dl. This is congruent with previous reports in South Africa,^48^ Tanzania,^46^ Malaysia^14^ and SSA.^49^ Anemia during TB and HIV infection impairs the immune response, hastening disease progression and leading to treatment failure, loss to follow-up and death.^50^ It also increases viral load, decreases CD4 count, progresses HIV to advanced stages and contributes to overall mortality.

The current review indicates that patients with fair/poor ART adherence experienced threefold the hazard for death than those with good ART adherence among TB and HIV co-infected patients (pooled HR: 3.11). Studies in Cameroon and Ethiopia support this report.^4,46^ This might be related to caregivers lack of commitment, low education levels and forgetfulness about daily ART drugs, which may lead to poor outcomes; this and non-adherence results in drug resistance, treatment failure and increased healthcare costs.

In contrast to previous SRM findings,^14,48,51,52^ particularly in resource-limited settings such as Nigeria and Malaysia,^14^ reported findings in the Caribbean, Central and South America and West Africa^53^ have shown a significant association between declining CD4 counts (≤200 cells/mL) and mortality in TB and HIV co-infection. However, in the current study, CD4 counts below the threshold and treatment failure did not show an association with the mortality of co-infected children. This discrepancy may be attributed to methodological variations, including a limited number of studies with sample size constraints.

## Strengths and limitations

Sensitivity and subgroup analyses were conducted to explore heterogeneity among the eligible studies. In addition to English language publications, articles in other languages were considered for inclusion. Despite these strengths, this review has its limitations, such as the exclusion of qualitative studies and limited access to primary studies from all regions of Ethiopia. Furthermore, some included articles had small sample sizes that could have impacted the accuracy of the pooled estimates.

## Conclusions

The review shows high mortality rates among individuals co-infected with TB and HIV. The contributing factors included poor ART adherence, advanced WHO staging, no IPT and anemia. Scaling up IPT, ART counseling and early treatment for anemia are key. Collaborative efforts and close patient follow-ups are crucial to reduce mortality and improve survival rates.

## Supplementary Material

ihaf085_Supplemental_Files

## Data Availability

The datasets used and analyzed during the current study will be made available upon reasonable request to the corresponding author via email contact.

## References

[1] Kegne TW, Anteneh ZA, Bayeh TL, et al. Survival rate and predictors of mortality among TB-HIV co-infected patients during tuberculosis treatment at public health facilities in Bahir Dar City, northwest Ethiopia. Infect Drug Resist. 2024;17:1385–95. 38618582 10.2147/IDR.S446020 PMC11015844

[2] WHO . Global Tuberculosis Report. 2023. Available at: https://www.who.int/teams/global-tuberculosis-programme/tb-reports/global-tuberculosis-report-2023 [accessed September 2024].

[3] Bizuneh FK, Bizuneh TK, Masresha SA, et al. Tuberculosis-associated mortality and risk factors for HIV-infected population in Ethiopia: a systematic review and meta-analysis. Front Public Health. 2024;12:1386113. 39104893 10.3389/fpubh.2024.1386113 PMC11298472

[4] Kebede Bizuneh F, Tsegaye D, Negese Gemeda B, et al. Proportion of active tuberculosis among HIV-infected children after antiretroviral therapy in Ethiopia: A systematic review and meta-analysis. PLoS Glob Public Health. 2024;4(8):e0003528. 39093892 10.1371/journal.pgph.0003528 PMC11296650

[5] Gesesew H, Tsehayneh B, Massa D, et al. Predictors of mortality in a cohort of tuberculosis/HIV co-infected patients in Southwest Ethiopia. Infect Dis Poverty. 2016; 5(1)109. 27915999 10.1186/s40249-016-0202-1 PMC5137204

[6] Girma D, Abita Z, Shifera N, et al. Incidence rate of tuberculosis among HIV infected children in Ethiopia: systematic review and meta-analysis. BMC Pediatric. 2024;24(1):363. 10.1186/s12887-024-04819-7 PMC11127285 38790006

[7] GBD 2021 Causes of death . Global burden of 288 causes of death and life expectancy decomposition in 204 countries and territories and 811 subnational locations, 1990-2021: a systematic analysis for the Global Burden of Disease Study 2021. Lancet. 2024;403(10440):2100–32. 38582094 10.1016/S0140-6736(24)00367-2 PMC11126520

[8] Uppal A, Rahman S, Campbell JR, et al. Economic and modeling evidence for tuberculosis preventive therapy among people living with HIV: A systematic review and meta-analysis. PLoS Med. 2021;18(9):e1003712. 34520463 10.1371/journal.pmed.1003712 PMC8439468

[9] Schutz C, Barr D, Andrade BB, et al. Clinical, microbiologic, and immunologic determinants of mortality in hospitalized patients with HIV-associated tuberculosis: A prospective cohort study. PLoS Med. 2019;16(7):e1002840. 31276515 10.1371/journal.pmed.1002840 PMC6611568

[10] Mandalakas AM, Kay AW, Bacha JM, et al. Tuberculosis among children and adolescents at HIV treatment centers in sub-Saharan Africa. Emerg Infect Dis. 2020;26(12):2933–43. 33219815 10.3201/eid2612.202245 PMC7706926

[11] Judd A, Doerholt K, Tookey PA, et al. Morbidity, mortality, and response to treatment by children in the United Kingdom and Ireland with perinatally acquired HIV infection during 1996-2006: planning for teenage and adult care. Clin Infect Dis. 2007;45(7):918–24. 17806062 10.1086/521167

[12] Ford N, Matteelli A, Shubber Z, et al. TB as a cause of hospitalization and in-hospital mortality among people living with HIV worldwide: a systematic review and meta-analysis. J Int AIDS Soc. 2016;19(1):20714. 26765347 10.7448/IAS.19.1.20714 PMC4712323

[13] Naidoo K, Gengiah S, Yende-Zuma N, et al. Mortality in HIV and tuberculosis patients following implementation of integrated HIV-TB treatment: Results from an open-label cluster-randomized trial. E Clin Med. 2022;44:101298. 10.1016/j.eclinm.2022.101298 PMC8850328 35198922

[14] Ismail I, Bulgiba A. Predictors of death during tuberculosis treatment in TB/HIV co-infected patients in Malaysia. PLoS One. 2013;8(8):e73250. 23951346 10.1371/journal.pone.0073250 PMC3741191

[15] Mollel EW, Todd J, Mahande MJ, et al. Effect of tuberculosis infection on mortality of HIV-infected patients in northern Tanzania. Tropical Med Health. 2020;48(1):26. 10.1186/s41182-020-00212-z PMC7184680 32355448

[16] Chu R, Mills EJ, Beyene J, et al. Impact of tuberculosis on mortality among HIV-infected patients receiving antiretroviral therapy in Uganda: a prospective cohort analysis. AIDS Res Therapy. 2013;10(1):19. 10.1186/1742-6405-10-19 PMC3716897 23849301

[17] Belay GM, Wubneh CA. Childhood tuberculosis treatment outcome and its association with HIV co-infection in Ethiopia: a systematic review and meta-analysis. Trop Med Health. 2020;48:7. 32099521 10.1186/s41182-020-00195-x PMC7027074

[18] Azanaw MM, Derseh NM, Yetemegn GS, et al. Incidence and predictors of tuberculosis among HIV patients after initiation of antiretroviral treatment in Ethiopia: a systematic review and meta-analysis. Trop Med Health. 2021;49(1):18. 33632342 10.1186/s41182-021-00306-2 PMC7905193

[19] Edessa D, Adem F, Hagos B, et al. Incidence and predictors of mortality among persons receiving second-line tuberculosis treatment in sub-Saharan Africa: a meta-analysis of 43 cohort studies. PLoS One. 2021;16(12):e0261149. 34890421 10.1371/journal.pone.0261149 PMC8664218

[20] Eshetie S, Gizachew M, Alebel A, et al. Tuberculosis treatment outcomes in Ethiopia from 2003 to 2016, and impact of HIV co-infection and prior drug exposure: a systematic review and meta-analysis. PLoS One. 2018;13(3):e0194675. 29554144 10.1371/journal.pone.0194675 PMC5858841

[21] Belay GM, Yehualashet FA, Ewunetie AW, et al. Pediatrics HIV-positive status disclosure and its predictors in Ethiopia: A systematic review and meta-analysis. PeerJ. 2022;10:e13896. 36032949 10.7717/peerj.13896 PMC9415365

[22] Kebede F, Kebede T, Kebede B, et al. Time to develop and predictors for incidence of tuberculosis among children receiving antiretroviral therapy. Tuberc Res Treat. 2021;2021:6686019. 34812290 10.1155/2021/6686019 PMC8605917

[23] Juszkiewicz K, Jarosz MJ, Wloszczak-Szubzda A, et al. Effectiveness of tuberculosis prophylaxis in patients with HIV/AIDS—retrospective analysis of data from Almaty, Kazakhstan, 2010-2015. AAEM. 2020;27(4):695–701. 33356080 10.26444/aaem/118611

[24] Hu FH, Tang XL, Ge MW, et al. Mortality of children and adolescents co-infected with tuberculosis and HIV: a systematic review and meta-analysis. AIDS. 2024;38(8):1216–27. 38499478 10.1097/QAD.0000000000003886

[25] Kassaw A, Asferie WN, Azmeraw M, et al. Incidence and predictors of tuberculosis among HIV-infected children after initiation of antiretroviral therapy in Ethiopia: A systematic review and meta-analysis. PLoS One. 2024;19(7):e0306651. 38968268 10.1371/journal.pone.0306651 PMC11226042

[26] Soeters HM, Napravnik S, Patel MR, et al. The effect of tuberculosis treatment on virologic and CD4+ cell count response to combination antiretroviral therapy: a systematic review. AIDS. 2014;28(2):245–55. 24072197 10.1097/01.aids.0000434936.57880.cd PMC4512169

[27] Gelaw YA, Assefa Y, Soares Magalhaes RJ et al. TB and HIV Epidemiology and Collaborative Service: evidence from Ethiopia, 2011–2015. HIV/AIDS. 2020;12:839–47. 10.2147/HIV.S284722 PMC7721114 33299356

[28] Page MJ, McKenzie JE, Bossuyt PM et al. The PRISMA 2020 statement: an updated guideline for reporting systematic reviews. BMJ. 2021;372:n71. 33782057 10.1136/bmj.n71 PMC8005924

[29] Barker TH, Hasanoff S, Aromataris E et al. The revised JBI critical appraisal tool for the assessment of risk of bias for cohort studies. JBI Evid Synth. 2025;23(3):441–53. 39177422 10.11124/JBIES-24-00103

[30] Egger M, Smith GD, Schneider M, et al. Bias in meta-analysis detected by a simple, graphical test. BMJ. 1997;315(7109):629–34. 9310563 10.1136/bmj.315.7109.629 PMC2127453

[31] Lin L, Chu H. Quantifying publication bias in meta-analysis. Biometrics. 2018;74(3):785–94. 29141096 10.1111/biom.12817 PMC5953768

[32] Borenstein M, Hedges LV, Higgins JP, et al. A basic introduction to fixed-effect and random-effects models for meta-analysis. Res Synth Methods. 2010;1(2):97–111. 26061376 10.1002/jrsm.12

[33] Duval S, Tweedie R, Nonparametric A. “Trim and fill” method of accounting for publication bias in meta-analysis. J Am Statist Assoc. 2000;95(449):89–98.

[34] Atalell KA, Birhan Tebeje N, Ekubagewargies DT. Survival and predictors of mortality among children co-infected with tuberculosis and human immunodeficiency virus at University of Gondar Comprehensive Specialized Hospital, Northwest Ethiopia. A retrospective follow-up study. PLoS One. 2018;13(5):e0197145. 29787596 10.1371/journal.pone.0197145 PMC5963769

[35] Chanie ES, Gelaye GA, Tadesse TY, et al. Estimation of lifetime survival and predictors of mortality among TB with HIV co-infected children after test and treat strategies launched in Northwest, Ethiopia, 2021; a multicentre historical follow-up study. PLoS One. 2021;16(12):e0258964. 34932563 10.1371/journal.pone.0258964 PMC8691625

[36] Dawit Z, Abebe S, Dessu S, et al. Incidence and predictors of mortality among children co-infected with tuberculosis and human immunodeficiency virus at public hospitals in Southern Ethiopia. PLoS One. 2021;16(6):e0253449. 34191846 10.1371/journal.pone.0253449 PMC8244885

[37] Gemechu J, Gebremichael B, Tesfaye T, et al. Predictors of mortality among TB-HIV co-infected children attending anti-retroviral therapy clinics of selected public hospitals in southern, Ethiopia: retrospective cohort study. Arch Public Health. 2022;80(1):11. 34983618 10.1186/s13690-021-00713-1 PMC8728901

[38] Nigussie J, Kassa M, Halefom G et al. Predictors of mortality among children co-infected with tuberculosis and human immunodeficiency virus in region, North Ethiopia, retrospective follow-up study. Biomed J Sci Tech Res. 2021;39(1):31026–34.

[39] Tsegaye D, Wude S, Kebede T, et al. Epidemiological survival pattern, risk factors, and estimated time to develop tuberculosis after test and treat strategies declared for children living with human immune deficiency virus. Indian J Tuberculosis. 2023;70(Suppl 1):S89–99. 10.1016/j.ijtb.2023.05.008 38110268

[40] Mukuku O, Mutombo AM, Kakisingi CN, et al. Tuberculosis and HIV co-infection in Congolese children: risk factors of death. Pan African Med J. 2019;33:326. 10.11604/pamj.2019.33.326.18911 PMC6815491 31692828

[41] Kebede F, Kebede B, Kebede T, et al. Effect of Isoniazid preventive therapy on the incidence of tuberculosis among seropositive children attending HIV/AIDS care in two General hospitals, Northwest Ethiopia, 2021. J Trop Med. 2021;2021:9996953. 34545289 10.1155/2021/9996953 PMC8448989

[42] Holmberg V, Soini H, Kivela P, et al. Epidemiology and outcome of HIV patients in Finland co-infected with tuberculosis 1998-2015. BMC Infect Dis. 2019;19(1):264. 30885144 10.1186/s12879-019-3890-x PMC6423794

[43] Nachega JB, Kapata N, Sam-Agudu NA, et al. Minimizing the impact of the triple burden of COVID-19, tuberculosis and HIV on health services in sub-Saharan Africa. Int J Infect Dis. 2021;113:S16–21. 33757874 10.1016/j.ijid.2021.03.038 PMC7980520

[44] Woldegeorgis BZ, Asgedom YS, Gebrekidan AY, et al. Mortality and its predictors among human immunodeficiency virus-infected children younger than 15 years receiving antiretroviral therapy in Ethiopia: a systematic review and meta-analysis. BMC Infect Dis. 2024;24(1):471. 38702591 10.1186/s12879-024-09366-1 PMC11069260

[45] Mollel EW, Todd J, Mahande MJ, et al. Effect of tuberculosis infection on mortality of HIV-infected patients in Northern Tanzania. Trop Med Health. 2020;48:26. 32355448 10.1186/s41182-020-00212-z PMC7184680

[46] Mugusi SF, Ngaimisi E, Janabi MY, et al. Risk factors for mortality among HIV-positive patients with and without active tuberculosis in Dar es Salaam, Tanzania. Antivir Ther. 2012;17(2):265–74. 22293579 10.3851/IMP1956

[47] Geremew D, Endalamaw A, Negash M, et al. The protective effect of isoniazid preventive therapy on tuberculosis incidence among HIV positive patients receiving ART in Ethiopian settings: a meta-analysis. BMC Infect Dis. 2019;19(1):405. 31077133 10.1186/s12879-019-4031-2 PMC6511123

[48] Mabunda TE, Ramalivhana NJ, Dambisya YM. Mortality associated with tuberculosis/HIV co-infection among patients on TB treatment in the Limpopo province, South Africa. African Health Sci. 2014;14(4):849–54. 10.4314/ahs.v14i4.12 PMC4370064 25834493

[49] Edessa D, Sisay M, Dessie Y. Unfavorable outcomes to second-line tuberculosis therapy among HIV-infected versus HIV-uninfected patients in sub-Saharan Africa: a systematic review and meta-analysis. PLoS One. 2020;15(8):e0237534. 32797110 10.1371/journal.pone.0237534 PMC7428180

[50] Gezae KE, Hagos K, Gebreslassie AA. Severity and determinants of anemia in TB/HIV coinfected adults at Mekelle, Ethiopia: hospital based retrospective study. J Trop Med. 2023;2023:5555030. 37234694 10.1155/2023/5555030 PMC10208761

[51] Rossetto M, Brand É M, Rodrigues RM, et al. Factors associated with hospitalization and death among TB/HIV co-infected persons in Porto Alegre, Brazil. PLoS One. 2019;14(1):e0209174. 30601842 10.1371/journal.pone.0209174 PMC6314623

[52] Tshitenge S, Ogunbanjo GA, Citeya A. A mortality review of tuberculosis and HIV co-infected patients in Mahalapye, Botswana: Does cotrimoxazole preventive therapy and/or antiretroviral therapy protect against death? African J Primary Health Care Family Med. 2018;10(1):e1–e5. 10.4102/phcfm.v10i1.1765 PMC6244457 30456967

[53] Ebonyi AO, Oguche S, Agbaji OO, et al. Mortality among pulmonary tuberculosis and HIV-1 co-infected Nigerian children being treated for pulmonary tuberculosis and on antiretroviral therapy: a retrospective cohort study. Germs. 2016;6(4):139–50. 28053917 10.11599/germs.2016.1099 PMC5187755

