# Supplementary material for: Risk factors and mortality rates for children co-infected with HIV and TB in Ethiopia: a systematic review and meta-analysis
Source: Int Health. 2025 Aug 21;18(2):145–53. doi: 10.1093/inthealth/ihaf085 (PMC13017473; doi:10.1093/inthealth/ihaf085)
Supplement: ihaf085_Supplemental_Files [file ihaf085_supplemental_files.zip › S2=Artciles searching strategy from PubMed.docx]

| Searching strategies in key words and Mesh terms | **Pumed/MEDLINE database** |
| --- | --- |
| **#1** | “Incidence” “OR” “Incidence Density rate” |
| **#2** | Mortality OR survival OR "Survival"[Mesh] OR "Mortality"[Mesh] |
| **#3** | Predictors OR “associated factors” OR determinants OR “risk factors” |
| **#4** | “associated factors” OR determinants OR “risk factors” |
| **#5** | “Tuberculosis-Human Immunodeficiency Virus co- infection” OR “TB-HIV co-infection” OR “TB-HIV co-infected patients” OR “TB and HIV infected children ” OR “TB-HIV co-infected adolescents” OR TB-HIV co-infected Pediatrics” OR “ Co-infected infants for TB and HIV” |
| **#6** | “Ethiopia” OR” Tigray” Addis Ababa” |
| **Final** | (((("Incidence"[Mesh] OR proportions[tw] OR "incidence rate" OR "incidence density" OR "time to death" [tw] OR burden[tw] OR magnitude[tw] AND ((ffrft[Filter]) AND (humans[Filter]) AND (English[Filter]))) AND (mortality[tw] OR survival[tw] OR "Survival"[Mesh] OR "Mortality"[Mesh] AND ((ffrft[Filter]) AND (humans[Filter]) AND (English[Filter])))) AND (predictors[tw] OR "associated factors" OR determinants OR "risk factors" AND ((ffrft[Filter]) AND (humans[Filter]) AND (English[Filter])))) AND ("Tuberculosis-Human Immunodeficiency Virus co- infection" OR "TB-HIV co-infection" OR "TB-HIV co-infected patients" OR "TB-HIV co-infected persons" OR "TB-HIV co-infected adolescents" AND ((ffrft[Filter]) AND (humans[Filter]) AND (English[Filter])))) AND (Ethiopia AND ((ffrft[Filter]) AND (humans[Filter]) AND (English[Filter]))) AND ((ffrft[Filter]) AND (humans[Filter]) AND (English[Filter])) |

S1 Table: Article searching search strategies and entry terms from international electronic data bases for mortality and Predictors of TB and HIV co-infected children in Ethiopia using SMR is
