# Supplementary material for: Risk factors and mortality rates for children co-infected with HIV and TB in Ethiopia: a systematic review and meta-analysis
Source: Int Health. 2025 Aug 21;18(2):145–53. doi: 10.1093/inthealth/ihaf085 (PMC13017473; doi:10.1093/inthealth/ihaf085)
Supplement: ihaf085_Supplemental_Files [file ihaf085_supplemental_files.zip › S3=JBI Checklist.docx]

**Table2: Quality appraisal result of included studies by** Joanna Briggs Institute (JBI) checklist

| **Full first Author name** | **Quality assessment questions** | | | | | | | | | | |  |  |  |
| --- | --- | --- | --- | --- | --- | --- | --- | --- | --- | --- | --- | --- | --- | --- |
|  | Two group employments at beginning | Exposure status ascertainment | Random selection of employment | Non response biases management | Data collection | Cases definition given | Strategies for in-complete follows- up management | Follow up time adequate | Confounding controlling | Outcome ascertainment at end of follow up | Was statically analysis correctly | Quality score | Quality status | Overall appraisal |
| **Jifare Gemechu et.al (1)** | Y | Y | Y | Y | Y | Y | Y | Y | Y | Y | Y | **3** | Low risk | Included |
| **Zinabu Dawit et.al (2)** | Y | Y | Y | Y | UN | Y | Y | Y | Y | y | Y | **3** | Low risk | Moderate |
| **Ermias sisay et.al (3)** | Y | Y | Y | Y | UN | Y | Y | Y | Y | Y | Y | **3** | Low risk | Included |
| **Jemberu Nigussie et.al(4)** | UN | Y | Y | Y | Y | Y | Y | Y | Y | Y | Y | **3** | Low risk | Moderate |
| **kindalem Attale et.al(5)** | Y | Y | Y | Y | UN | Y | Y | Y | Y | Y | Y | **3** | Low risk | Included |
| **Dejen Tsegaye et.al(6)** | Y | Y | Y | Y | Y | Y | Y | Y | Y | Y | Y | **3** | Low risk | Included |

Key: Y=yes, N=no, UC=unclear, Q=Question

Grading of risk ofbiases for included articles was scored as follow

When score of articles is from 8 up to 11= will had (>75%) score, good quality , low risk of bias and included

When score of articles from 5 up to 7 = will had (50 to 75%) ,moderate quality ,low risk &artciles will be included

When score of artciles is from 3 upt o 5 = will had 25%to 50 %), high risk of biases and artciles will be not included

**References**

1. Gemechu J, Gebremichael B, Tesfaye T, Seyum A, Erkalo D. Predictors of mortality among TB-HIV co-infected children attending anti-retroviral therapy clinics of selected public hospitals in southern, Ethiopia: retrospective cohort study. Archives of public health = Archives belges de sante publique. 2022;80(1):11.

2. Dawit Z, Abebe S, Dessu S, Mesele M, Sahile S, Ajema D. Incidence and predictors of mortality among children co-infected with tuberculosis and human immunodeficiency virus at public hospitals in Southern Ethiopia. PLOS ONE. 2021;16(6):e0253449.

3. Chanie ES, Gelaye GA, Tadesse TY, Feleke DG, Admas WT, Molla Alemu E, et al. Estimation of lifetime survival and predictors of mortality among TB with HIV co-infected children after test and treat strategies launched in Northwest, Ethiopia, 2021; a multicentre historical follow-up study. PloS one. 2021;16(12):e0258964.

4. Jemberu Nigussie MKGHHH. Predictors of Mortality Among Children Co-Infected with Tuberculosis and Human Immunodeficiency Virus in Region, North Ethiopia, Retrospective Follow-

Up Study. Biomed J Sci & Tech Res | BJSTR MSID006252. 2021.

5. Atalell KA, Birhan Tebeje N, Ekubagewargies DT. Survival and predictors of mortality among children co-infected with tuberculosis and human immunodeficiency virus at University of Gondar Comprehensive Specialized Hospital, Northwest Ethiopia. A retrospective follow-up study. PLOS ONE. 2018;13(5):e0197145.

6. Tsegaye D, Wude S, Kebede T, Adane S, Shumet T, Kebede F. Epidemiological survival pattern, risk factors, and estimated time to develop tuberculosis after test and treat strategies declared for children living with human immune deficiency virus. Indian Journal of Tuberculosis. 2023;70:S89-S99.
